# Still Not Solved: A Call for Renewed Focus on User-Centered Teleoperation Interfaces

**DOI:** 10.3389/frobt.2022.704225

**Published:** 2022-03-29

**Authors:** Daniel J. Rea, Stela H. Seo

**Affiliations:** ^1^ Faculty of Computer Science, University of New Brunswick, Fredericton, NB, Canada; ^2^ Department of Social Informatics, Kyoto University, Kyoto, Japan

**Keywords:** teleoperation, interfaces, user-centered design, user-centered teleoperation, literature review, human-robot interaction

## Abstract

Teleoperation is one of the oldest applications of human-robot interaction, yet decades later, robots are still difficult to control in a variety of situations, especially when used by non-expert robot operators. That difficulty has relegated teleoperation to mostly expert-level use cases, though everyday jobs and lives could benefit from teleoperated robots by enabling people to get tasks done remotely. Research has made great progress by improving the capabilities of robots, and exploring a variety of interfaces to improve operator performance, but many non-expert applications of teleoperation are limited by the operator’s ability to understand and control the robot effectively. We discuss the state of the art of user-centered research for teleoperation interfaces along with challenges teleoperation researchers face and discuss how an increased focus on human-centered teleoperation research can help push teleoperation into more everyday situations.

## 1 Introduction

Teleoperated robots, robots controlled at a distance, are already used in a number of situations where it is unsafe for humans to be physically present, such as search-and-rescue (Casper and Murphy, 2003; BBCClick, 2017; Peskoe-Yang, 2019) after natural disasters (Settimi et al., 2014; Norton et al., 2017; Tadokoro, 2019), scientific research such as moving underwater (Delmerico et al., 2019), working in space (Schilling et al., 1997), or making critical inspection and repairs (Buonocore, 2021; González et al., 2021; Koh et al., 2021). However, with a small number of exceptions, we see no remotely operated robots in use by the general public, whether it is at work or in their personal space. When we look to existing teleoperation situations–often robots in extreme situations–we find that teleoperation, even when performed by expert operators, is still an extremely difficult task, requiring multiple specialized operators for a single robot (Norton et al., 2017; Murphy and Tadokoro, 2019). Despite such human resources, it is still difficult to perform even basic collision avoidance, and this operation difficulty increases operator stress (Norton et al., 2017; Murphy and Tadokoro, 2019). If experts struggle to teleoperate robots remotely, then it is likely that the average person, even using simpler robots, would also struggle.

Teleoperation research has long noted that one of the bottlenecks to teleoperation performance is the operator’s abilities, which can be limited by the technology itself, such as camera field of view (Endsley, 1988; Yanco et al., 2004; Chen et al., 2007; Lin et al., 2007; Niemeyer et al., 2016). In light of this, a long-term effective strategy for research is to create robots and interfaces that specifically reduce these limiting factors of the robot, such as adding additional camera views (Saakes et al., 2013; Rakita et al., 2019a), leveraging multiple robots semi-autonomously (Rakita et al., 2019b; Coelho et al., 2021), or inventing new ways to control robots (Escolano et al., 2012; Choi et al., 2018). This research has resulted in numerous new techniques that can benefit numerous teleoperation scenarios. Despite the progress in these areas, teleoperation remains difficult.

In this paper, we review core challenges in teleoperation interface design and recent systematic surveys and find that teleoperation performance, especially for non-experts, is still hindered by the operator and the interface they use to control and monitor the robot. We found less user-centered work, instead focusing on improving and augmenting teleoperation technology to mitigate its weaknesses. User-centered work, in contrast, started from the abilities and needs of the operator and built interfaces with them in mind. Showcasing the potential of this approach, we highlight classic and recent examples for human-centered and non-expert teleoperation interface design [e.g., for manipulation (Herlant et al., 2016; Kent et al., 2017; Li et al., 2017; Rakita et al., 2017), shared autonomy (Jain et al., 2015; Aronson et al., 2018; Rakita et al., 2019b; Jeon et al., 2020; Nicolis, 2020), camera control (Rakita et al., 2018; Rakita et al., 2019a), and social and psychological interfaces (Rea et al., 2017a; Rea and Young, 2018; Rea and Young, 2019a; Rea et al., 2020; Seo et al., 2020; Valiton and Li, 2020)], we call for teleoperation and robot researchers broadly to use more additional advanced applications of user-centered practices by starting with user-driven solutions in addition to existing technically-driven approaches.

We further argue that engage with user-centered problems in teleoperation in a variety of applications, the field should focus more on simple everyday applications for non-expert users. We found that these seemingly simple tasks such as turning a door knob are still surprisingly difficult for modern teleoperation approaches, and we describe broad research directions for making user-centered interfaces as well as user-focused methods. Existing user-centered teleoperation research demonstrates that our call is a complimentary approach to traditional teleoperation research that has simply received less attention, but nevertheless has the potential for impact. This paper emphasizes and broadens other recent calls for increased focus on human factors (Murphy and Tadokoro, 2019), general usability (George et al., 2015), and information visualization (Szafir and Szafir, 2021). These directions can help bring teleoperated robots into daily life to improve productivity, accessibility, and more.

## 2 Core Problems in Teleoperation Interaction

Using recent systematic surveys as a base [e.g., general telerobotics (Niemeyer et al., 2016), interfaces for teleoperated manipulators (Young and Peschel, 2020), or field robotics (Liu and Nejat, 2013; Delmerico et al., 2019; Murphy and Tadokoro, 2019)], we informally surveyed teleoperation interface research in recent years, as well as more influential work from the past 2 decades. We targeted the keywords of “teleoperation,” “interaction,” “interface,” and “user-centered,” in our journal and conference searches, and excluded work that was primarily engineering, algorithmic, or expert use-cases such as teleoperated space or surgery robots. We found most work focused on aiding two major user-centered problems in both expert and non-expert teleoperation: situation awareness and robot control. Together, these problems create a significant cognitive burden on the operator, making teleoperation difficult, regardless of the task being done with the robot. We briefly describe and discuss these major problems, and highlight some of the larger approaches we found. In particular, we found some research highlighting the user-centered nature of these problems, and successes for user-centered solutions.

### 2.1 Visualization of the Remote Data for Teleoperation Awareness

The term, situation awareness, emerged from aviation psychology to describe the pilot’s understanding of tactical flight operations (Durso and Gronlund, 1999), but it is applicable broadly to any cognitive activity and information processing, including teleoperation (Durso and Gronlund, 1999; Yanco and Drury, 2004a; Endsley, 2015). Situation awareness, in teleoperation, is an operator’s ability to perceive, understand, and reason about the environment around them and around their remote robot (Endsley, 1988; Rakita et al., 2017; Rakita et al., 2018; Nicolis, 2020), which requires the operator to process a large amount of robot sensor data from the remote environment in real-time (Goodrich et al., 2013).

#### 2.1.1 Sensors for Situation Awareness

**FIGURE 1:**
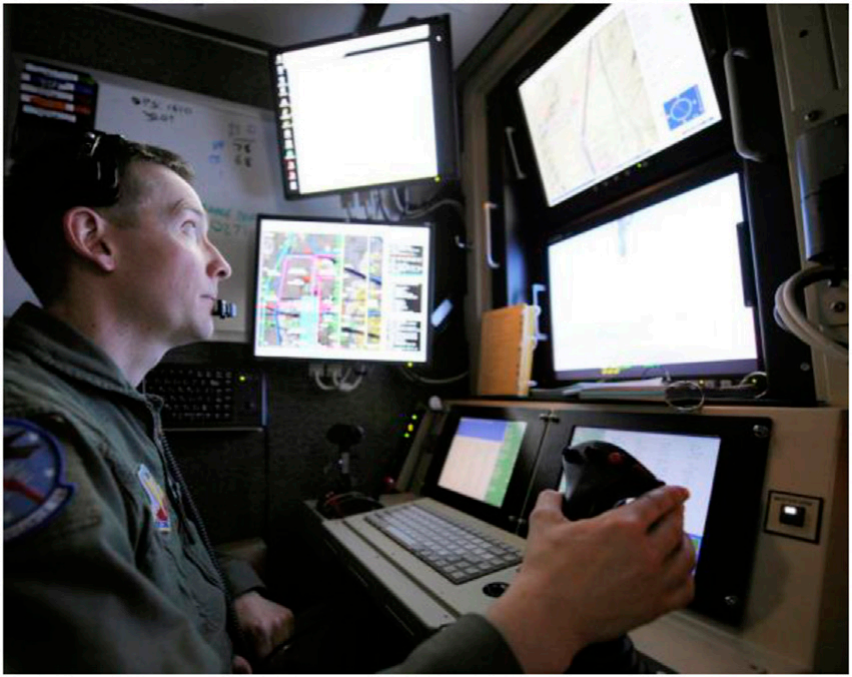
Cockpit for remote drone teleoperation. Pilots need to process numerous sensors while handling mission tasks with multiple controls in stressful situations. (wikipedia.org/wiki/File:6th_Reconnaissance_Squadron_-_Operator.jpg, public domain).

**FIGURE 2:**
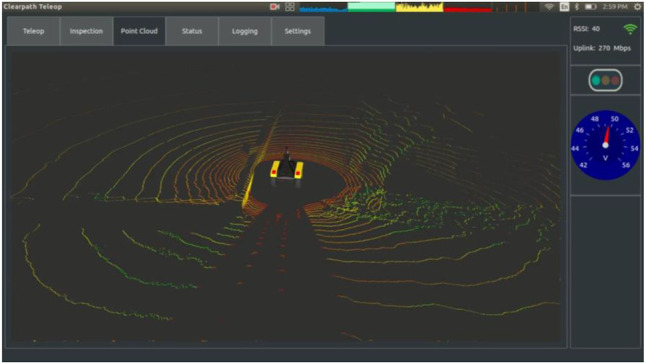
A teleoperation interface from Clearpath Robotics. This tablet interface contains numerous sensor data streams which are accessed through mode switching via the top buttons in a tab-like interface. This interface reduces information load for the operator, but makes accessing all information quickly more difficult due to the need to switch tabs (source: Clearpath Robotics).

Research has been improving an operator’s ability to build and maintain a high level of situation awareness for decades (Endsley, 1988; Endsley, 2016; Niemeyer et al., 2016). A general first-step approach is to add sensors to enable the robot to provide some information for the operator to perceive the remote space and make better decisions [e.g., (Endsley, 1988; Endsley, 2016; Yanco and Drury, 2004a), see Figures 1, 2]. Sensors may add perceptual abilities that people typically do not have, such as depth sensors that can see in the dark (Mast et al., 2015), or sonar that detects nearby objects (Nielsen et al., 2007). Additional sensors may instead simply add more advantageous data, such as egocentric cameras that provide a better detailed view, or exocentric cameras to provide a better sense of where the robot is in its environment (Saakes et al., 2013; Seo et al., 2017a). Entire other robots may be added to provide more abilities and viewpoints (Saakes et al., 2013; Rakita et al., 2019a; Coelho et al., 2021).

Adding more information via sensors, however, does not always help the operator’s situation awareness, as people can only pay attention to a certain amount of information at once (Drury et al., 2003; Endsley, 2016; Norton et al., 2017). Instead of additional sensors, the user may be encouraged to perform robot actions to actively observe the environment [active perception (Bajcsy et al., 2018)]. This adds additional controls and perception needs to teleoperation, creating more cognitive load. The challenge of increasing cognitive load with robot capability is an on-going research topic in human-robot interaction (George et al., 2015; Delmerico et al., 2019; Murphy and Tadokoro, 2019). Research has therefore focused on reducing cognitive load for building and maintaining situation awareness to improve overall teleoperator performance.

#### 2.1.2 Visualizations for Increasing Situational Awareness

To gain the benefits of additional sensors without increasing cognitive load, research is actively developing new techniques to visualize sensor data (Drury et al., 2003; Yanco and Drury, 2004b). A general trend is to process more raw data for the operation, and then create a display of those results, that is, easy for the operator to understand and reason about. For example, interfaces can highlight points of interest in ways that naturally draw the operator’s interest (Rea et al., 2017b), leverage people’s existing knowledge to summarize an off-screen’s object’s position and distance (Seo et al., 2017b), or combine multiple mediums like sound or haptics [multi-modal interfaces such as (Hacinecipoglu et al., 2013; Suarez Fernandez et al., 2016; Nicolis, 2020; Seo et al., 2020)]. Making robot sensor visualizations is not always obvious as sensors may detect qualities that are hard to visualize with a screen or speakers [such as haptic and force data (Reveleau et al., 2015; Nicolis, 2020)]. Interfaces like those described here—and many others (Nielsen et al., 2007; Yang et al., 2015; Seo et al., 2017a)—that fuse and interpret sensor data are essentially performing situation awareness processing for the operator, instead of having the operator analyze data or separate visualizations to come to conclusions themselves. How to produce such visualizations is difficult and an on-going topic in teleoperation, requiring more research in information visualization (Szafir and Szafir, 2021).

### 2.2 Robot Control Interfaces

In addition to creating and maintaining situation awareness from sensor data, the operator must make decisions quickly for their tasks and provide commands to a robot using the available controls. Understanding how to control a robot is also difficult for operators. This may be adjusting the robot’s body pose, such as moving a multi-jointed robot arm, or to help drive a robot through an environment. Control itself consists of many problems, including situation awareness, simplify control complexity, choosing levels of autonomy for an action, or dealing with physical problems like latency. In general, the control scheme must be clear so that operators can understand and reason about how to command a robot to complete a task they may have—known as a gulf of execution that the operator must cross with the help of good interaction design (Norman et al., 1986). We found research typically focuses on one of two problems: how the input is provided by the operator, and what level of automation the robots behaviors use.

#### 2.2.1 Level of Automation for Controls

A fundamental choice for robot control interfaces is how autonomous the actions will be. Historically, this has been almost no automation, with operators manually controlling each motor [e.g., early arms seen in (Singh et al., 2013) or remote-controlled vehicles]. However, in recent years, research has increasingly focused on introducing more semi-autonomous behaviors, enabling simple inputs to result in complex behaviors (Singh et al., 2013; Herlant et al., 2016; Kent et al., 2017; Li et al., 2017; Rakita et al., 2017; Rakita et al., 2019a; Nicolis, 2020; Young and Peschel, 2020). In addition, high levels of automation are vital to assist teleoperators in managing multiple robots while performing their tasks (Squire and Parasuraman, 2010; Wong and Seet, 2017). Automation enables the operator to think less about the robot’s computers, sensors, and joints, and more as a tool that can accomplish tasks, reducing cognitive load.

#### 2.2.2 Freeing Operators With Semi-Autonomous Controls

While there are clear benefits to semi-autonomous controls, there are tradeoffs and other problems introduced. For example, consider if the operator may define some level of goal (destination, pose, action, etc.), and then the robot autonomously proceeds partially or completely to that goal [e.g., (Quigley et al., 2004; Singh et al., 2013; Tsui et al., 2013)]. Once commands have been given, there is time while the robot proceeds which an operator can use to deal with other tasks [e.g., (Olsen and Wood, 2004; Glas et al., 2012)].

**FIGURE 3:**
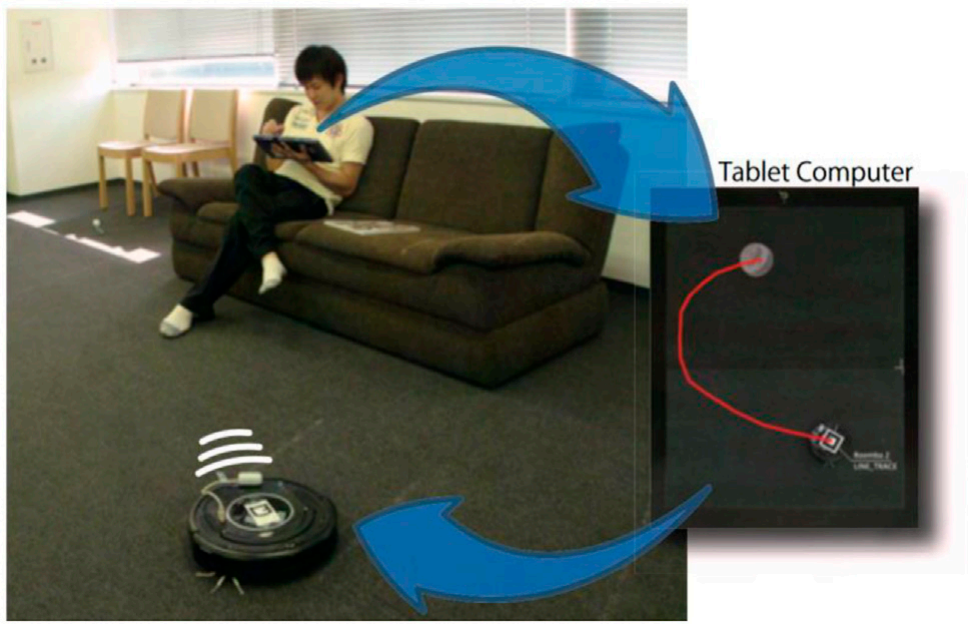
A sketch-based control interface for a robot that overlays commands in an overhead view of the real world to aid control and understandability of the robot’s future actions. From Sakamoto et al. (2009), with permission*.*

However, algorithms may be imperfect and the real world is dynamic, and so it may be necessary for the operator to provide more input during a task, such as help a kinematics simulator predict what position would be best to grip an option with a robotic arm (Leeper et al., 2012). Another potential drawback is that while attending other tasks, operators must maintain situation awareness of the teleoperation task, or reacquire it upon returning to the robot, potentially delaying task completion and adding workload to the operator (Donald et al., 2015). An operator may wish to edit or cancel an existing command in real-time, adding more complexity to the interaction. Because of the benefits for operator multitasking, and long-term planning of robot actions [e.g., (Liu et al., 2011; Sakamoto et al., 2009), see Figure 3], these problems for semi-autonomous teleoperation remain under active research, with research inventing new algorithms for autonomous behaviors and investigating their user acceptance [e.g., (Dragan and Srinivasa, 2013; Mehr et al., 2016; Javdani et al., 2018; Reddy et al., 2018; Brooks and Szafir, 2019)].

#### 2.2.3 Robot and Mixed Initiative Controls

In addition to executing actions semi-autonomously, robots may also take actions by themselves (machine initiative) instead of waiting for operator commands [human initiative (Kortenkamp et al., 1997; Gombolay et al., 2017)]. This is particularly suited to be used in multiple robot teleoperation scenarios where one operator controls multiple robots [e.g., (Kolling et al., 2012; Glas et al., 2012)]. However, any autonomous action by the robot again threatens to break an operator’s situation awareness and can also be confusing to the user if it is unclear the robot is taking initiative or a result of an operator command (mixed initiative systems). Such shared autonomy is a promising Frontier for improved usability and under active research [e.g., (Dragan and Srinivasa, 2013; Jain et al., 2015; Mehr et al., 2016; Aronson et al., 2018; Javdani et al., 2018; Reddy et al., 2018; Brooks and Szafir, 2019; Jeon et al., 2020), discussed later]. Recent research has shown the consistency and transparency in these robot-initiated actions is key to a better user experience (Small et al., 2018). Therefore, even with high levels of robot autonomy, we still need to consider the operator’s user experience when creating teleoperation interfaces.

#### 2.2.4 Input Strategies for Teleoperation

Even controlling a single robot is a challenging task that taxes an operator’s cognitive resources (Steinfeld et al., 2006); seemingly basic tasks such as navigating a single, wheeled robot around a space are difficult enough that researchers have invented interfaces that aim to reduce the overhead required for a teleoperator in such a situation [e.g., (Barber et al., 2010; Young et al., 2011; Singh et al., 2013)].

#### 2.2.5 Input Strategies for Non-Expert Teleoperators

Some strategies specifically targeting non-expert users (Kent et al., 2017; Li et al., 2017; Rakita et al., 2017; Rakita et al., 2019b; Jeon et al., 2020), such as employing well-known control metaphors [e.g., a dog leash for a robot companion (Young et al., 2011)], visualizing the results of a command (Singh et al., 2013), using intuitive controls such as sketching paths in an image of the environment (Sakamoto et al., 2009; Sugiura et al., 2010; Liu et al., 2011) (see Figure 3). These earlier works leverage user-centered design—the interface designs are rooted in familiar ways of acting and thinking (behavioral and cognitive psychology, human factors). We note that these controls simplify or reduce the degrees of freedom in the interface that the user has to explicitly think about (e.g., they simply move a pen instead of working with multi-button or multi-axis controls for 2D or 3D movement). With this approach of making interfaces more approachable, simpler, and familiar, the general public are more likely to find the controls usable than controls built for engineers or programmers (Singh et al., 2013; Delmerico et al., 2019).

#### 2.2.6 Dynamic Control Granularity

Another strategy is to allow flexible levels of control. For example, an operator may need to define a precise path through an environment or grip an object at a certain angle; in these cases it is common to have complete control over robot movements with specialized interfaces designed for one robot’s capabilities [e.g., (Sakamoto et al., 2009; Hashimoto et al., 2011; Glas et al., 2012)]. However, complex controls can make some actions, especially common actions, tiring to manually perform repeatedly. For these situations, one strategy is to combine those common but complex commands into single actions that are easy to invoke (Barber et al., 2010; Jain et al., 2015; Jeon et al., 2020)—once again simplifying the control space the user needs to think about. By understanding the tasks operators wish to complete with a robot, the interfaces can be made more manual or more automated to ease teleoperation.

While promising and demonstrably effective, we see little of these advances in modern teleoperation in our daily lives. It is possible that even further usability advances in feedback interfaces are needed to make teleoperation more accessible to the general public.

### 2.3 Robot Awareness of the Operator

An even less studied aspect of situation awareness that relates to controls is the robot’s (and the teleoperation interface’s) awareness of the operator. In other words, to properly execute commands and display appropriate feedback, teleoperation interfaces and robots should consider the environment and how commands serve the operator’s goals (Endsley, 1988). A robot may also be aware of an operator’s state, such as by user modelling—algorithmically predicting what a person is thinking, feeling, or will do (Basu et al., 2017; Jain and Argall, 2020; Jeon et al., 2020), or by directly monitoring the user [e.g., (Jia et al., 2014; Aronson et al., 2018)]. For example, control can be simplified by guessing operator intention to move to a space or pose and using automation to move towards that goal [e.g., (Gopinath et al., 2017; Aronson et al., 2018; Jain and Argall, 2020)], or the robot may understand a command but modify feedback to the operator to encourage better collision avoidance (Hacinecipoglu et al., 2013). Interfaces could even present information to create a specific state in the operator to affect future teleoperation (Rea and Young, 2019a; Rea et al., 2020).

Robots can also consider *how* an operator thinks a task should be completed, such as asking the operator to give input when an algorithm cannot decide between several courses of action [e.g., (Leeper et al., 2012)]. The robot should also be aware if the operator is distracted or busy with other tasks if input is needed, and use strategies such as taking intelligent automated actions until the operator is free [e.g., (Glas et al., 2012)], or the robot could use interfaces to draw operator attention quickly to important areas without distracting them too much (Rea et al., 2017b). Guessing operator intentions can be used to assess how much the operator has paid attention interface feedback, and could create an estimate of trust in operator commands.

Drawbacks and open challenges include how to integrate such machine initiative or shared autonomy actions in a way, that is, not disliked or confusing to operators (Wang and Lewis, 2007; Norton et al., 2017). Thus, by understanding the operator’s state and goals, a robot can autonomously adapt commands to a dynamic world in an intelligent way.

The key idea is for the teleoperation system itself to consider not just the operator’s commands, but their state—a user-aware interface—in order to help the operator’s situation awareness and control accuracy. In other words, the operator is the center of the interface design. Because of this, we believe that even more user-centered designs and methodologies than are currently used are necessary for improving teleoperation.

## 3 User-Centered Practices and Teleoperation

In our survey, we noted a trend for research to focus on solving core problems through additional robot capabilities and interface components, or addressing technical weaknesses (sensors, algorithms, etc.). We also found solutions driven by user needs and interface design in early and recent teleoperation research; these user-centered approaches show great promise, but we found fewer of these compared to technology-driven work. We discuss these and other recent works while asking–Why is teleoperation still so difficult?

### 3.1 Expert Interfaces for Usability Problems

Teleoperation has benefited from an increase in robot capabilities and robot-centered research, with improvements in reliability, robustness, sensing, traversal, and more (Delmerico et al., 2019; Murphy and Tadokoro, 2019). There was a general focus on expert users in highly technical, dangerous, and high-pressure situations, such as search and rescue use cases, bomb disposal and firefighting. In these cases, it is critical that the operator build an accurate mental picture of the situation quickly and control the robot successfully. Surveys noted that because the operator is so preoccupied with safe and careful operation, they often work in teams of multiple stakeholders (operators, engineers, decision-makers, etc.), that direct and advise the operator at an objective-level, rather than a robot level. This creates communication challenges between the types of operators, and researchers have noted they may each require a bespoke interface for optimal performance (Murphy and Tadokoro, 2019).

This leads to most modern teleoperation interfaces being expert interfaces—systems that assume extensive training and deep knowledge of a specialized system or application for good performance (Turchetti et al., 2012)^1^. These systems, due to their very specific user base, circumvent the need for common usability and learnability standards, often allowing or excusing increased information density, and complex, manual control. In this light, multiple operators may simply be one workaround to the usability difficulties of these systems. Both older (Endsley, 1988; Drury et al., 2003; Yanco et al., 2004; Chen and Thropp, 2007; Chen et al., 2011) and recent research (George et al., 2015; Murphy and Tadokoro, 2019; Szafir and Szafir, 2021), however, has identified that even these expert systems still have a need to incorporate and learn from basic HCI research (information density, learnability, layout, etc.) to further aid experts during teleoperation and decisions making, and going even further by developing and leveraging more advanced and specialized HCI areas like information visualization (Szafir and Szafir, 2021).

### 3.2 A Call for Additional Focus in User-Centered Teleoperation

While acknowledging that user-centered research has and continues to be an active subfield of teleoperation research [e.g., for manipulation (Jain et al., 2015; Herlant et al., 2016; Kent et al., 2017; Li et al., 2017; Rakita et al., 2017; Aronson et al., 2018; Rakita et al., 2019b), integrating automation (Jain et al., 2015; Rakita et al., 2019b; Jeon et al., 2020; Nicolis, 2020), or better sensor use (Rea et al., 2017b; Rakita et al., 2019a; Rea and Young, 2019a; Rea et al., 2020; Seo et al., 2020)], based on our survey we call for more teleoperation researchers *to engage with teleoperation as a fundamentally user-driven domain* that should be approached with *user-centered processes*—a user evaluation alone, performed after development is only the beginning. Our goal should be that even everyday, non-expert people should be able to use complex robots with advanced manipulators, sensors, and movement abilities to improve everyday situations, and that *they should be included throughout the design and development process*. Even experts benefit from better user experience, usability, and learnability [e.g., in both general software (Dziubak et al., 2016) and robotics (Huang and Cakmak, 2017)], and user-focused improvements will lead to more accessible teleoperation in a variety of applications.

Recent research notes that teleoperation is fundamentally multitasking—doing a task with a robot, while figuring out how to accomplish that task safely and efficiently with the robot (Murphy and Tadokoro, 2019). Improving the fundamental basics of an interface (human factors, presentation, etc.) has long been known as an important factor in teleoperation (Yanco et al., 2004; Chen et al., 2007; Chen et al., 2011; George et al., 2015) but either sufficient improvement has yet to be made, or these basic HCI principles are insufficient on their own, perhaps due to the inherent complexities and difficulties of teleoperation (Norton et al., 2017; Norton et al., 2018; Murphy and Tadokoro, 2019; Szafir and Szafir, 2021). We propose that to conduct this user-focused teleoperation, more research should focus on general users in everyday applications for teleoperation, as it removes the ability to rely on expertise and technical ability.

To aid in this refocusing towards user-centered teleoperation, we highlight several applications of teleoperated robots that are not as extreme as the focus of many field robotics studies, note their unique problems that come from a more general user base, and motivate several future approaches for more research. We conclude that the shared nature of these problems with the core teleoperation problems described above suggests that teleoperation in general can progress by also investigating these simpler, more constrained applications, which in turn could provide new techniques and avenues for improvements in extreme robotics as well.

## 4 Everyday Applications of Teleoperation—Still Not Solved

We have been arguing that teleoperation is fundamentally difficult from a usability perspective. To this end, we believe that researching interfaces tailored to everyday non-expert applications and users is important, as it provides similar research challenges in a simpler and more tractable testbed to progress fundamental usability issues in teleoperation. In fact, there have long been researchers that study everyday applications of teleoperation [e.g., (Niemeyer et al., 2016; Kent et al., 2017; Rakita et al., 2017)] where they encounter and study similar problems to more typical search-and-rescue robotics.

Telepresence can enter our daily lives in any situation a person needs to be in a physical space but cannot for any reason (health, distance, money, visas, etc.). Telepresence technologies has been used for children to attend school (Tsui et al., 2013), remote business meetings (Lee and Takayama, 2011), and more (Kristoffersson et al., 2013; Tsui et al., 2015). However, the core problems of teleoperation, difficult for experts, can be even more challenging for non-expert users. While well-developed commercial products exist, telepresence robots (Neustaedter et al., 2016; Rae and Neustaedter, 2017) are far from a solved problems, where challenges emerge from the surprising complexities of everyday situations and the interfaces needed to successfully navigate them.

Many industries have some level of routine inspection and maintenance needs that could be done with teleoperated robots. Some industries have well-defined and structured tasks and environments and tasks that could be leveraged as a simpler environment to develop better interfaces. Like more difficult applications of robots, these applications require robot navigation in constrained spaces, detailed inspection with multiple advanced sensors, logging, and reporting, and sometimes engaging in repair work using carefully controlled manipulators (Buonocore, 2021; González et al., 2021; Koh et al., 2021). These operators are often specialized in the industry the robot is used in, but not necessarily familiar with robotics or similar technology themselves. Thus, industrial teleoperation should benefit from increased user-centered research to aid these non-expert users while also acting as a simpler testbed for more complex types of teleoperation.

Teleoperated robots also have the potential to provide help in everyday situations, accessibility, and assist with ageing-in-place—assistive telerobotics (Goodrich et al., 2013; Jain et al., 2015; Tsui et al., 2015; Okamura and Tanaka, 2016; Jeon et al., 2020). For people who may have difficulties with mobility, strength, a comprised immune system (e.g., in a pandemic), or simply need assistance in a task, teleoperated robots could help by making tasks easier, reducing risk of injury or exposure to diseases. With improved interface design, teleoperated robots may improve feelings of efficacy, satisfaction, and independence of the home operators. One promising existing example of this technology is robotic wheelchairs with manipulators, which are not remote but still face typical core teleoperation challenges (Al-qaysi et al., 2018; Dragomir et al., 2021). These users may need extra help or have trouble using interfaces due to special needs, but designing for such users can improve customizability and accessibility for all users [e.g., (Jain et al., 2015; Jeon et al., 2020)].

Teleoperated robots may also increasingly become a form of everyday recreation [e.g., drone racing (Pfeiffer and Scaramuzza, 2021)]. The sport requires interfaces to support operators to drive safely but quicly while maintaining awareness in a highly dynamic environment. Drone racing is thus a useful real-world scenario to develop and test interfaces that help even everyday operators to leverage their skills to perceive, navigate, and interact with the environment in a highly dynamic situation. It further doubles as a safer test application for search and rescue robotics as fast-paced and difficult teleoperation situation, but with fewer serious consequences.

Looking back to core problems with teleoperation, we can see they all remain in everyday situations—achieving situation awareness with various sensors, and controls for navigation and manipulation. These tasks seem simple compared to the challenge of disaster response and search-and-rescue, but these “simple” everyday applications of teleoperation are still difficult from a usability perspective. We propose teleoperation requires research that focuses on the interaction design, and to develop more best practices and guidelines for user-centered interface design in teleoperation**.**

### 4.1 Considerations and Opportunities for Everyday User-Centered Teleoperation

The core user-centered teleoperation problems continue to be important to research from a systems perspective. However, with a user-centered approach, the research goals should shift to learnability, usability, and a better user experience, which can themselves increase operator performance, efficiency, and decision-making ability. We noted that everyday teleoperation applications provide good related and safer real-life situations to test and develop interfaces in. Here we discuss some main differences and opportunities in these applications: the operators, the robots, and the situations the robots are operated in.

#### 4.1.1 The Operators

The operators are often experts in some field, but are not roboticists, engineers, or computer scientists. Thus, interfaces should not assume operators understand how to interpret a point cloud, how a differential drive works, or how kinematics affects how an arm can move; such topics will need to be avoided, hidden, or taught quickly but carefully in an interface. Operators may also not even be experts in their own specific robots, using them only a few times a year or less to do a maintenance task at their workplace. Everyday operators will also be more sensitive to user experience: since they are usually not required to use the robot, the robot must compete with the familiarity and ease of doing a task in a non-robotic way. They may even need additional accessibility considerations. The need to consider the lack of robot knowledge, familiarity, accessibility, and patience with poor user experience is not new in teleoperation research [e.g., (Drury et al., 2003; Chen et al., 2007; Sakamoto et al., 2009; Jain et al., 2015)], but we argue they need to become a core design consideration, as the experience and needs of operators differ heavily—additional use of user-centered research methods will be beneficial.

#### 4.1.2 The Robots

The robots everyday people use may not be as advanced, robust, accurate, or powerful as those used by experts in extreme search-and-rescue situations, and this is potentially beneficial. For example, the commercial Double 3 telepresence robot does not have hundred-meter-reaching sensors, may not survive falling over or outdoor use, and does not move very quickly—we know how to build a more capable robot. However, these constraints came about from user-centered design: fewer robot features make creating a simple interface easier, it enables the robot to be built to suit a specific use case (e.g., indoor office or school use), and keep costs down for accessibility. In other words, a capable robot is not necessarily a better robot for a given user or application (Rea et al., 2017a). Leveraging these constraints and designing robots specifically with user needs in mind throughout the engineering process for more telepresence applications is an opportunity to improve robot accessibility and translate to better interfaces.

#### 4.1.3 The Environment

The environments these robots are used in also may present specific challenges. For example, robots may be in dynamic or crowded spaces (a telepresence robot at a conference), or in an unknown and non-structured environment (a doctor tele-visiting a patient at their home). However, many environments are much more structured and regular than what search and rescue robots may be able to expect: factories have a well-known and predictable environment for inspection and maintenance robots, grocery stores have organized, easy-to-see-and-reach shelves of goods for a robot being used to pick up groceries, or public spaces like school or malls have a pre-defined environment. In addition, the tasks needed to be performed in these spaces may be completely or mostly defined in advance, such as a robot for routine maintenance. By understanding the user’s needs in the task and environment, robots can be better designed to help in the most common cases.

#### 4.1.4 Error Tolerance

Finally, while there are exceptions, many teleoperation applications are in situations that have some level of fault tolerance—delays will not result in lost lives, and minor collisions may only result in an apology and no harm. Thus, non-expert interfaces have an opportunity to help people learn how the mistakes they encountered came to be, and help avoid them in the future. This suggests that common performance metrics like number of errors may need to be rethought, and interfaces should explicitly be designed and tested expecting a certain level of operator error.

These considerations share the idea of simplification: less pre-supposed robot knowledge, simpler robots, simpler, safer, and more structured environments, and smaller costs for error. These simplifications may help make interface research more practical, while staying grounded in real-world problems, before extending to more difficult applications. While none of these problems are new or unknown [see active research such as (Herlant et al., 2016; Kent et al., 2017; Li et al., 2017; Rakita et al., 2017; Rakita et al., 2018; Rakita et al., 2019a; Rakita et al., 2019b; Jeon et al., 2020; Nicolis, 2020; Rea et al., 2020)], we call for additional attention to these problem areas as they are well suited for studying general user needs and perspectives.

## 5 Current User-Centered Approaches

We emphasize that the current systems and usability work being done in teleoperation is valuable—there are still hard robotics problems that would benefit usability if they were solved, and fundamental human factors work is still necessary as robotic platforms and new interaction methods (e.g., augmented or virtual reality) arise. In particular, we want to highlight how some existing work, some mentioned previously in Section 2, is user-focused and how those processes and techniques benefit teleoperation technologies.

### 5.1 Interfaces for Feedback

Research has developed many techniques over decades for displaying sensor data in an intuitive way, but the situation awareness problem is still unsolved (Norton et al., 2017; Norton et al., 2018; Delmerico et al., 2019; Murphy and Tadokoro, 2019). This is partly because new types of robot platforms and sensors have appeared, and it has become common for robots to have multiple sensors and cameras at once, increasing the operator’s potential mental workload. If expert users struggle to interpret multiple sources of advanced sensor data, then there is a further need for simplified and easy to interpret data displays for non-expert operators.

Recent calls for bringing in advanced information visualization techniques acknowledge this, and is an important future approach to improving teleoperation (Szafir and Szafir, 2021). Some of these techniques even leverage new display technologies like augmented and virtual reality to explore new ways to present interfaces in a natural way (Nielsen et al., 2007; Labonte et al., 2010; Hedayati et al., 2018; González et al., 2021). We would encourage additional focus on visualizations that consider less experienced users, who may not understand or be familiar with the limitations of sensor or data types, who will likely be slower with any technique, and may be harder to train due to lack of foundational knowledge in engineering, or computer science.

Other recent surveys have noted that fundamental and basic human-computer interaction principles, such as limiting simultaneous information and considering interface layout, use of colors, and more are important (Niemeyer et al., 2016; Delmerico et al., 2019; Murphy and Tadokoro, 2019; Young and Peschel, 2020; Szafir and Szafir, 2021). Our survey agrees, and we encourage the development of additional user-centered teleoperation guidelines with additional and more in-depth user-driven solutions targeting non-expert applications.

### 5.2 Controls

Controls have also improved in terms of usability. Both aspects of the core problems discussed in Section 2 have improved with user-focused methods: the physical hardware of the controller, and the way software can be used to add additional controls or abstract actions.

#### 5.2.1 Simplifying With Abstraction and Automation

We observed a general trend for human-centered systems to keep controls simple by providing higher-level controls compared to traditional teleoperation systems (Sakamoto et al., 2009; Leeper et al., 2012; Ochiai et al., 2014; Kent et al., 2017), such as a point-and-click system navigation, with more complex controls being hidden with modes and menus. For more complex robots, the basic controls will often be modal, with the most common controls all accessible with video game or 3D haptic controllers, with perhaps advanced or more specific manual controls requiring something like a keyboard and mouse. However, there are many more advances for complex telemanipulation (Herlant et al., 2016; Kent et al., 2017; Li et al., 2017; Rakita et al., 2017; Rakita et al., 2018; Rakita et al., 2019a; Nicolis, 2020).

We also saw a trend for more abstract controls with automation, which can help non-expert operators with less experience. The key approach here is leveraging some level of autonomy to enable the operators to think at a task level (e.g., “grab this”, “move there”, “stop and inspect on the left”), rather than needing to reason about robot morphology, motors, or other low-level robot factors (Rea et al., 2020). This can relieve the workload of the operator [e.g., Section 2.2.2 (Dragan and Srinivasa, 2013; Mehr et al., 2016; Javdani et al., 2018; Reddy et al., 2018; Brooks and Szafir, 2019)], though it may also create new user-centered problems related to initiative and transparency. This research is on-going and is necessary to both experts, and non-experts. In fact, being unable to rely on expertise may require even more clever displays of information and streamlined controls.

#### 5.2.2 Simplifying With Modal Interfaces

We note some successes with modal inputs - the system’s state changes how an input results in an action. For example, a joystick may normally move the robot forward and back, but the operator could enter a “manipulation mode” where the joystick instead moves a robot arm forward and back. Traditionally in human-computer interaction, modes are considered less usable for general software due to mistaking which mode the system is (Sarter and Woods, 1995) (mode error).

The common alternative for complex systems like telerobotics which often have many degrees of freedom inherently, however, is to just have a complex interface with many widgets, buttons, and controls (Yanco et al., 2015; Norton et al., 2017). The example modal control above is one method of enabling a smaller set of inputs to cover a broader range of robot actions. While this increases the possibility of mode error, well designed modal controls could simplify the controls space enough to make a net usability gain. Thus, more user centered work is required to gracefully enable high degree of freedom control to simple interfaces with potentially limited inputs.

As an example, we note that mode switching is commonly used in video games as a way to circumvent controllers with limited degrees of freedom to control a complex avatar. While video games are not necessarily simple to control, they are evidence that good user (or player)-centered designs can mitigate the drawbacks of modal designs and limit mode errors. We encourage this approach teleoperation designs, as teleoperation and video games have been shown to share many design similarities (Richer and Drury, 2006; Rea, 2020). Looking at the academic research of games usability, it further suggests that teleoperation may have its own set of usability guidelines that may differ from general software, encouraging further exploration of fundamental user-centered teleoperation research.

## 6 Research Directions for User-Centered Teleoperation

We are calling for a renewed user-centered focus to teleoperation interfaces, especially for everyday applications for non-expert users. We acknowledge that there has always been user-centered research in teleoperation, however our survey found limited engagement with this approach, focusing more often on technical solutions to user-based problems in expert applications. We view additional user-centered research as complimentary to existing systems-focused research in teleoperation and it will help operators take full advantage of the hardware and algorithms being developed today. In fact, many of these recommendations still require significant technical contributions to enable these user-centered approaches. We have highlighted state of the art successful techniques that are already demonstrating the power of this approach and point the way for future directions in a user-centered teleoperation.

We acknowledge the considerable overlap between the following high-level research directions, but we recommend future works focus on:

### 6.1 Help the User Do

Robot control is a large and general problem. However, there is already evidence that consistent, reliable controls that are intuitive and engaging to use while also accomplishing high-level actions can improve teleoperation. Future interfaces for robot control should aim for the following goals:

#### 6.1.1 More Abstract Controls

A key trend we see in controls is abstraction–enabling operators to think more at a conceptual level than a robot or hardware level. Leveraging partial automation such as shared autonomy (Dragan and Srinivasa, 2013; Mehr et al., 2016; Javdani et al., 2018; Reddy et al., 2018; Brooks and Szafir, 2019) or other forms of automation can enable operators to think at the task level, rather than at the robot level. Manual modes should be placed behind menus or modes for more rare, dexterous tasks.

#### 6.1.2 Better Experiences—Consistent, Reliable, and Transparent Controls

A user needs to be able to predict how a command will be performed by the robot (consistency). When a command is executing, succeeds, fails, or needs user input, the system should communicate this to the user (transparency). These, along with other guidelines, create good user experiences (Forlizzi and Battarbee, 2004; Hassenzahl and Tractinsky, 2006), and enable an operator to act with confidence, be engaged, satisfaction, and willingness to use again. We saw very little recent user experience-focused teleoperation work, but is known to be important to teleoperation (George et al., 2015), and interactive applications in general (Benyon, 2019).

#### 6.1.3 Model the User to Better Interpret Commands

To be user-centered, the system should first understand the user. We suggest teleoperation systems further explore user monitoring (with sensors) and user modelling (predict how they are feeling and thinking) and adjust the interface and interpretation of commands accordingly. For example, the robot could detect a non-expert user is nervous, and ignore sudden erratic commands that are caused by nerves, or detect a tired operator and slow them down while reducing information displays (cognitive load) to help them think clearer.

### 6.2 Help the User Understand

Situation awareness is not simply about providing more information, it is about combining that information and visualizing it in a way that helps the user think and act effectively which is not straightforward in remote teleoperation. While this has been noted in other works (Seo et al., 2017a; Rea et al., 2017b; Seo et al., 2017b; Rea and Young, 2018; Rea and Young, 2019b; Szafir and Szafir, 2021), our own literature search corroborates this goal, and should be emphasized and be applied broadly to teleoperation. Mental workload is a fundamental metric for evaluating interface designs as workload has been strongly linked to teleoperation performance, and so research continues to target interfaces that reduce it, as well as improve other performance metrics (e.g., awareness) while adding minimal additional workload.

#### 6.2.1 Leverage Human Psychology to Help People Process Information Naturally

People naturally process information in certain ways, for example, movement on screen can automatically draw a user’s attention (Teng et al., 2013) and people automatically process information encoded in social interfaces (Feldmaier et al., 2017; Rea et al., 2020). This incurs a lower cognitive load than a multi-step rational analysis, for example, a multi-dimensional graph, which can be slow or need training non-expert operators may not have. Thus, we recommend visualizations that replicate how operators naturally process information (Rea et al., 2017b; Rea and Young, 2019a; Rea et al., 2020) (familiar representations such as faces, maps, etc.), which can be used to sidestep difficult engineering problems like computer vision, making the operator and robot as a sort of team (Mingyue Ma et al., 2018).

#### 6.2.2 Create Understanding for Users and Avoid Displaying Raw Data

Modern teleoperation systems are often designed as expert-user interfaces, and so commonly display large amounts of information. However, for better teleoperation for all users, we recommend interfaces should add *knowledge* instead of information, by processing raw data and presenting it in a form, that, is more relevant and useful to users (Szafir and Szafir, 2021). Alternatively, the system should predict and only display relevant information to an operator, perhaps by leveraging known properties of the task, environments, or users. This could limit workload and increase non-expert operator ability, while still allowing deeper and expert interfaces to be present in menus, hidden for when needed.

#### 6.2.3 Encourage Active Perception

People can build a better understanding by actively asking questions and exploring a space through action—active perception, or thinking through embodiment (Klemmer et al., 2006; Wainer et al., 2006; Bajcsy et al., 2018). An interface could encourage a user to move to a space, use a robot sensor or manipulator in a new way to understand the environment, and generally explore through interaction instead of simply thinking about the robot and sensor outputs. This method can be tailored to guide non-expert users especially, who may feel more confident moving and interacting in a space like they naturally do.

### 6.3 Help the User Learn

Even with user-centered interfaces, some training is inevitable Software learning has suggested general approaches to reduce the need and cost of this training, and turn using the robot into a learning experience for less experienced users:

#### 6.3.1 Require Minimal Upfront Knowledge and Training

New designs should require minimal upfront specialized knowledge, and instead teach on-the-fly or leverage common designs that users can leverage from their everyday experiences (e.g., video games). If specialized interfaces are used, consider explaining them as they become relevant. For example, a telepresence system could detect the robot is looking at someone who is talking towards the robot, but that person’s face is not visible. This could activate a tutorial to move the robot’s camera to better see them and engage in conversation, teaching a new skill in an applied environment.

#### 6.3.2 Enable Review of Interfaces and Mistakes

Another goal to improve non-expert teleoperation is to provide avenues for building skill, maintaining skill, reviewing the interface, and preventing future mistakes, as mistakes will happen and can be more excusable in everyday situations. Thus, we recommend researching interfaces that help users understand *when* an error occurred, *how* it occurred, and even how to *prevent* it in the future. For example, if a user does make an error (a collision, or has to stop and reposition the robot, etc.), the system could explain parts of the interface that were meant to inform the user of the nearby object, or even show a replay of recent operation, pointing out where the mistake could have been prevented.

We note our research directions are themselves user-centered. When building a user interface, researchers should focus on what they want to aid operators with, what the outcomes should be, and include users in the design process (not just in the evaluation stage). Teleoperation is not only about making improving robot capabilities, it is also about improving people’s ability to complete tasks with robots.

Our goal in the medium-term is for comfortable single-robot non-expert operation. While the current multi-expert team standard in search and rescue teleoperation may maximize a robot’s lifesaving potential, everyday non-expert operators have relaxed performance requirements and penalties for mistakes. This provides opportunity to better explore how to reduce information intelligently, help semi-automate common robot tasks, and improve interface learning and training. Teams of expert operators may always be the most effective in critical situations, but striving for comfortable single-operation by non-experts can make robots more appealing and applicable to a variety of applications.

## 7 Conclusion: Why is Teleoperation Still So Hard?

Teleoperation research has made great progress over the decades, improving robots, reducing latency, improving basic interfaces, and more. However, despite cheaper, more capable robots and many applications that could benefit from teleoperation, teleoperation remains at the edges of expert and extreme use cases. We argue that this is in part because teleoperation is a fundamentally difficult task for operators, and more user-centered methods should be applied to research in all areas of teleoperation design, especially in the interface. We surveyed teleoperation papers and found progress on the core teleoperation problems of control and situation awareness, and recent surveys and techniques that demonstrate the benefits of user-centered design for teleoperation. We called for a renewed focus in broad, user-centered research goals to improve teleoperation interfaces in everyday applications for non-experts, and to develop better interfaces that leverage how operators understand, think about, and use teleoperated robots. This leads us to recommend that end-users should be included throughout the teleoperation research process, not just as a user study at the end of a project, and that experiments should take advantage of such end-users’ approachable everyday environments as experiment settings to test teleoperation technologies in the real world. The results of this research should complement the existing research approaches and benefit teleoperation as a whole.

## Footnotes

1
https://abcnews.go.com/Blotter/drone-stigma-means-skilled-pilots-controls-deadly-robots/story?id=23475968

## References

[B3] Al-qaysi Z. T. Zaidan B. B. Zaidan A. A. Suzani M. S. (2018). A Review of Disability EEG Based Wheelchair Control System: Coherent Taxonomy, Open Challenges and Recommendations. Comput. Methods Programs Biomed. 164, 221–237. 10.1016/j.cmpb.2018.06.012 29958722

[B5] Aronson R. M. Santini T. Kübler T. C. Kasneci E. Srinivasa S. Admoni H. (2018). “Eye-Hand Behavior in Human-Robot Shared Manipulation,” in Human-Robot Interaction, 4–13. 10.1145/3171221.3171287

[B6] Bajcsy R. Aloimonos Y. Tsotsos J. K. (2018). Revisiting Active Perception. Auton. Robot 42 (2), 177–196. 10.1007/s10514-017-9615-3 PMC6954017 31983809

[B7] Barber C. M. Shucksmith R. J. MacDonald B. Wünsche B. C. (2010). “Sketch-based Robot Programming,” in 25th International Conference of Image and Vision Computing New Zealand.

[B8] Basu C. Yang Q. Hungerman D. Singhal M. Dragan A. D. (2017). “Do You Want Your Autonomous Car to Drive like You?,” in Human-Robot Interaction, 417–425.

[B9] BBCClick (2017). Fukushima Disaster: The Robots Going where No Human Can. *October 18* .

[B10] Benyon D. (2019). Designing User Experience: A Guide to HCI, UX and Interaction Design. Pearson.

[B11] Brooks C. Szafir D. (2019). “Balanced Information Gathering and Goal-Oriented Actions in Shared Autonomy,” in 2019 14th ACM/IEEE International Conference on Human-Robot Interaction (HRI), Daegu, Korea (South), 11-14 March 2019, 85–94. 10.1109/HRI.2019.8673192

[B12] Buonocore Luca. R. (2021). Overview of the Robotic Service at CERN for Accelerator Maintenance.

[B13] Casper J. Murphy R. R. (2003). Human-robot Interactions during the Robot-Assisted Urban Search and rescue Response at the World Trade Center. IEEE Trans. Syst. Man. Cybern. B 33 (3), 367–385. 10.1109/tsmcb.2003.811794 18238185

[B14] Chen J. Y. C. Barnes M. J. Harper-Sciarini M. (2011). Supervisory Control of Multiple Robots: Human-Performance Issues and User-Interface Design. IEEE Trans. Syst. Man. Cybern. C 41 (4), 435–454. 10.1109/tsmcc.2010.2056682

[B15] Chen J. Y. C. Haas E. C. Barnes M. J. (2007). Human Performance Issues and User Interface Design for Teleoperated Robots. IEEE Trans. Syst. Man. Cybern. C 37 (6), 1231–1245. 10.1109/tsmcc.2007.905819

[B16] Chen J. Y. C. Thropp J. E. (2007). Review of Low Frame Rate Effects on Human Performance. IEEE Trans. Syst. Man. Cybern. A. 37 (6), 1063–1076. 10.1109/tsmca.2007.904779

[B17] Choi P. J. Oskouian R. J. Tubbs S. R. (2018). Telesurgery: Past, Present, and Future. Cureus 10 (5), 1–5. 10.7759/cureus.2716 PMC6067812 30079282

[B18] Coelho A. Sarkisov Y. Wu X. Mishra H. Singh H. Dietrich A. (2021). Whole-Body Teleoperation and Shared Control of Redundant Robots with Applications to Aerial Manipulation. J. Intell. Robotic Syst. Theor. Appl. 102, 1. 10.1007/s10846-021-01365-7

[B19] Delmerico J. Mintchev S. Giusti A. Gromov B. Melo K. Horvat T. (2019). The Current State and Future Outlook of rescue Robotics. J. Field Robotics 36 (7), 1171–1191. 10.1002/rob.21887

[B20] Donald F. Donald C. Thatcher A. (2015). Work Exposure and Vigilance Decrements in Closed Circuit Television Surveillance. Appl. Ergon. 47 (January 2016), 220–228. 10.1016/j.apergo.2014.10.001 25479991

[B21] Dragan A. D. Srinivasa S. S. (2013). A Policy-Blending Formalism for Shared Control. Int. J. Robotics Res. 32 (7), 790–805. 10.1177/0278364913490324

[B22] Dragomir A. Pană C. F. Cojocaru D. Manta L. F. (2021). “Human-Machine Interface for Controlling a Light Robotic Arm by Persons with Special Needs,” in 2021 22nd International Carpathian Control Conference (ICCC).

[B23] Drury J. L. L. Scholtz J. Yanco H. A. (2003). “Awareness in Human-Robot Interactions,” in SMC'03 Conference Proceedings. 2003 IEEE International Conference on Systems, Man and Cybernetics. Conference Theme - System Security and Assurance (Cat. No.03CH37483), Washington, DC, USA, 8-8 Oct. 2003, 912–918.

[B24] Durso F. T. Gronlund S. D. (1999). “Situation Awareness,” in Handbook of Applied Cognition. Editor Durso F. T. (John Wiley & Sons, Inc.), 283–314.

[B25] Dziubak V. Dubois P. Bunt A. Terry M. (2016). “Switter: Supporting Exploration of Software Learning Materials on Social Media,” in Proceedings of the 2016 ACM Conference on Designing Interactive Systems, 1209–1220.

[B26] Endsley M. R. (2016). Designing for Situation Awareness: An Approach to User-Centered Design. CRC Press.

[B27] Endsley M. R. (1988). “Design and Evaluation for Situation Awareness Enhancement,” in Proceedings of the Human Factors and Ergonomics Society Annual Meeting. Sage 32, 97–101.

[B28] Endsley M. R. (2015). Situation Awareness: Operationally Necessary and Scientifically Grounded. Cogn. Tech. Work 17 (2), 163–167. 10.1007/s10111-015-0323-5

[B29] Escolano C. Antelis J. M. Minguez J. (2012). A Telepresence mobile Robot Controlled with a Noninvasive Brain-Computer Interface. IEEE Trans. Syst. Man. Cybern. B 42 (3), 793–804. 10.1109/tsmcb.2011.2177968 22180512

[B30] Feldmaier J. Stimpfl M. Diepold K. (2017). “Development of an Emotion-Competent SLAM Agent,” in Proceedings of the Companion of the 2017 ACM/IEEE International Conference on Human-Robot Interaction (HRI '17). New York, NY: ACM.

[B31] Forlizzi J. Battarbee K. (2004). “Understanding Experience in Interactive Systems,” in Proceedings of the 2004 conference on Designing interactive systems processes, practices, methods, and techniques - DIS ’04, Cambridge MA USA, August 1 - 4, 2004, 261. 10.1145/1013115.1013152

[B32] George A. Christou G. Katsanos C. Xenos M. Hadzilacos T. (2015). Usability Guidelines for the Design of Robot Teleoperation: A Taxonomy. IEEE Trans. Human-Machine Syst. 45 (2), 256–262. 10.1109/THMS.2014.2371048

[B33] Glas D. F. Kanda T. Ishiguro H. Hagita N. (2012). Teleoperation of Multiple Social Robots. IEEE Trans. Syst. Man. Cybern. A. 42 (3), 530–544. 10.1109/tsmca.2011.2164243

[B34] Gombolay M. Bair A. Huang C. Shah J. (2017). Computational Design of Mixed-Initiative Human–Robot Teaming that Considers Human Factors: Situational Awareness, Workload, and Workflow Preferences. Int. J. Robotics Res. 36 (5–7), 597–617. 10.1177/0278364916688255

[B35] González C. Ernesto Solanes J. Muñoz A. Gracia L. Girbés-Juan V. Tornero J. (2021). Advanced Teleoperation and Control System for Industrial Robots Based on Augmented Virtuality and Haptic Feedback. J. Manufacturing Syst. 59 (February), 283–298. 10.1016/j.jmsy.2021.02.013

[B36] Goodrich M. A. Crandall J. W. Barakova E. (2013). Teleoperation and beyond for Assistive Humanoid Robots. Rev. Hum. Factors Ergon. 9 (1), 175–226. 10.1177/1557234x13502463

[B37] Gopinath D. Jain S. Argall B. D. (2017). Human-in-the-Loop Optimization of Shared Autonomy in Assistive Robotics. IEEE Robot. Autom. Lett. 2 (1), 247–254. 10.1109/lra.2016.2593928 30662953 PMC6335047

[B38] Hacinecipoglu A. Konukseven E. I. Bugra Koku A. (2013). “Evaluation of Haptic Feedback Cues on Vehicle Teleoperation Performance in an Obstacle Avoidance Scenario,” in 2013 World Haptics Conference, WHC (2013). IEEE.

[B39] Hashimoto S. Ishida A. Inami M. Igarash T. (2011). “TouchMe: An Augmented Reality Based Remote Robot Manipulation,” in International Conference on Artificial Reality and Telexistence. Osaka, Japan.

[B40] Hassenzahl M. Tractinsky N. (2006). User Experience - A Research Agenda. Behav. Inf. Techn. 25 (2), 91–97. 10.1080/01449290500330331

[B41] Hedayati H. Walker M. Szafir D. (2018). “Improving Collocated Robot Teleoperation with Augmented Reality,” in Human-Robot Interaction. ACM/IEEE, 78–86. 10.1145/3171221.3171251

[B42] Herlant L. V. Holladay R. M. Srinivasa S. S. (2016). “Assistive Teleoperation of Robot Arms via Automatic Time-Optimal Mode Switching,” in 2016 11th ACM/IEEE International Conference on Human-Robot Interaction (HRI), Christchurch, New Zealand, 7-10 March 2016, 35–42. 10.1109/HRI.2016.7451731 PMC6053067 30035277

[B43] Huang J. Cakmak M. (2017). “Code3: A System for End-To-End Programming of Mobile Manipulator Robots for Novices and Experts,” in Proceedings of the 2017 ACM/IEEE International Conference on Human-Robot Interaction, Vienna, Austria, 6-9 March 2017, 453–462.

[B44] Jain S. Farshchiansadegh A. Broad A. Abdollahi F. Mussa-Ivaldi F. Argall B. (2015). Assistive Robotic Manipulation through Shared Autonomy and a Body-Machine Interface. IEEE Int. Conf. Rehabil. Robot 2015, 526–531. 10.1109/ICORR.2015.7281253 26855690 PMC4737957

[B45] Jain S. Argall B. (2020). Probabilistic Human Intent Recognition for Shared Autonomy in Assistive Robotics. J. Hum.-Robot Interact. 9 (1), 1–23. 10.1145/3359614 PMC7233691 32426695

[B46] Javdani S. Admoni H. Pellegrinelli S. Srinivasa S. S. Bagnell J. A. (2018). Shared Autonomy via Hindsight Optimization for Teleoperation and Teaming. Int. J. Robotics Res. 37 (7), 717–742. 10.1177/0278364918776060

[B47] Jeon H. J. Losey D. P. Sadigh D. (2020). “Shared Autonomy with Learned Latent Actions,” in Robotics: Science and Systems. 10.15607/rss.2020.xvi.011

[B48] Jia Y. Xi N. Liu S. Wang Y. Li X. Bi S. (2014). Quality of Teleoperator Adaptive Control for Telerobotic Operations. Int. J. Robotics Res. 33 (14), 1765–1781. 10.1177/0278364914556124

[B49] Kent D. Saldanha C. Chernova S. (2017). “A Comparison of Remote Robot Teleoperation Interfaces for General Object Manipulation,” in Proceedings of the 2017 ACM/IEEE International Conference on Human-Robot Interaction, Vienna, Austria, 6-9 March 2017, 371–379. 10.1145/2909824.3020249

[B50] Klemmer S. R. Hartmann B. Takayama L. (2006). “How Bodies Matter,” in Proceedings of the 6th ACM conference on Designing Interactive systems - DIS ’06, University Park PA USA, June 26 - 28, 2006, 140. 10.1145/1142405.1142429

[B51] Koh K. H. Farhan M. Ching Yeung K. P. Ho Tang D. C. Yee Lau M. P. Cheung P. K. (2021). Teleoperated Service Robotic System for On-Site Surface Rust Removal and protection of High-Rise Exterior Gas Pipes. Automation in Construction 125, 103609. 10.1016/j.autcon.2021.103609

[B4] Kolling A. Nunnally S. Lewis M. (2012). “Towards Human Control of Robot Swarms,” in 2012 7th ACM/IEEE International Conference on Human-Robot Interaction (HRI), 89–96.

[B52] Kortenkamp D. Bonasso R. P. Ryan D. Schreckenghost D. (1997). Traded Control with Autonomous Robots as Mixed Initiative Interaction. AAAI Tech. Rep. 04, 89–94.

[B53] Kristoffersson A. Coradeschi S. Loutfi A. (2013). A Review of mobile Robotic Telepresence. Adv. Human-Computer Interaction 2013, 1–17. 10.1155/2013/902316

[B54] Labonte D. Boissy P. Michaud F. (2010). Comparative Analysis of 3-D Robot Teleoperation Interfaces with Novice Users. IEEE Trans. Syst. Man. Cybern. B 40 (5), 1331–1342. 10.1109/tsmcb.2009.2038357 20106745

[B55] Lee M. K. Takayama L. (2011). “Now, I Have a Body”: Uses and Social Norms for mobile Remote Presence in the Workplace,” in Proceedings of Human Factors in Computing Systems: CHI 2011. Vancouver, CA, 33–42.

[B56] Leeper A. Hsiao K. Ciocarlie M. Takayama L. Gossow D. (2012). “Strategies for Human-In-The-Loop Robotic Grasping,” in 2012 7th ACM/IEEE International Conference on Human-Robot Interaction (HRI), Boston, MA, USA, 5-8 March 2012, 1–8.

[B57] Li Zhi. Moran P. Dong Q. Shaw R. J. Hauser K. (2017). “Development of a Tele-Nursing mobile Manipulator for Remote Care-Giving in Quarantine Areas,” in 2017 IEEE International Conference on Robotics and Automation (ICRA), Singapore, 29 May-3 June 2017, 3581–3586.

[B58] Lin C. T. Chuang S. W. Chen Y. C. Ko L. W. Liang S. F. Jung T. P. (2007). EEG Effects of Motion Sickness Induced in a Dynamic Virtual Reality Environment. Annu. Int. Conf. IEEE Eng. Med. Biol. Soc. 2007, 3872–3875. 10.1109/IEMBS.2007.4353178 18002844

[B59] Liu K. Sakamoto D. Inami M. Igarashi T. Rouge B. (2011). “Roboshop : Multi-Layered Sketching Interface for Robot Housework Assignment and Management,” in Human Factors in Computing (CHI). ACM.

[B60] Liu Y. Nejat G. (2013). Robotic Urban Search and rescue: A Survey from the Control Perspective. J. Intell. Robot Syst. 72 (2), 147–165. 10.1007/s10846-013-9822-x

[B61] Mast M. Materna Z. Španěl M. Weisshardt F. Arbeiter G. Burmester M. (2015). Semi-Autonomous Domestic Service Robots: Evaluation of a User Interface for Remote Manipulation and Navigation with Focus on Effects of Stereoscopic Display. Int. J. Soc. Robotics 7 (2), 183–202. 10.1007/s12369-014-0266-7

[B62] Mehr N. Horowitz R. Dragan A. D. (2016). “Inferring and Assisting with Constraints in Shared Autonomy,” in Conference on Decision and Control, CDC. IEEE, 6689–6696.

[B63] Mingyue Ma L. Fong T. Micire M. J. Kim Y. K. Feigh K. (2018). Human-Robot Teaming: Concepts and Components for Design, 649–663. 10.1007/978-3-319-67361-5_42

[B64] Murphy R. R. Tadokoro S. (2019). User Interfaces for Human-Robot Interaction in Field Robotics. Springer International Publishing.

[B65] Neustaedter C. Gina V. Procyk J. Hawkins D. (2016). To Beam or Not to Beam: A Study of Remote Telepresence Attendance at an Academic Conference. Proc. ACM Conf. Comput. Supported Coop. Work, CSCW 27, 418–431. 10.1145/2818048.2819922

[B66] Nicolis D. (2020). A General Framework for Shared Control in Robot Teleoperation with Force and Visual Feedback. Springer Briefs in Applied Sciences and Technology, 119–131. Special Topics in Information Technology. 10.1007/978-3-030-32094-2_9

[B67] Nielsen C. W. Goodrich M. A. Ricks R. W. (2007). Ecological Interfaces for Improving mobile Robot Teleoperation. IEEE Trans. Robot. 23 (5), 927–941. 10.1109/tro.2007.907479

[B68] Niemeyer G. Preusche C. Hirzinger G. (2016). “Telerobotics,” in Springer Handbook of Robotics (Berlin, Heidelberg: Springer), 741–757. 10.1007/978-3-319-32552-1_43

[B69] Norman D. A. (1986). in Cognitive Engineering. Editors Norman D. A Draper S (Erlbaum Associates), 31–62. 10.1201/b15703-3

[B1] Norton A. Ober W. Baraniecki L. McCann E. Scholtz J. Shane D. (2017). Analysis of Human–Robot Interaction at the DARPA Robotics Challenge Finals. Int. J. Robot. Res. 36 (5-7), 483–513.

[B2] Norton A. Ober W. Baraniecki L. Shane D. Skinner A. Yanco H. (2018). "The DARPA Robotics Challenge Finals: Humanoid Robots To The Rescue," in Springer Tracts in Advanced Robotics Editors Spenko M. Buerger S. Iagnemma K. Shane D.. (Cham: Springer), 121.

[B70] Ochiai Y. Takemura K. Ikeda A. Takamatsu J. Ogasawara T. (2014). “Remote Control System for Multiple mobile Robots Using Touch Panel Interface and Autonomous Mobility,” in 2014 IEEE/RSJ International Conference on Intelligent Robots and Systems, 3272–3277. 10.1109/IROS.2014.6943017

[B71] Okamura E. Tanaka F. (2016). “A Pilot Study about Remote Teaching by Elderly People to Children over a Two-Way Telepresence Robot System,” in 2016 11th ACM/IEEE International Conference on Human-Robot Interaction (HRI), Christchurch, New Zealand, 7-10 March 2016, 489–490. 10.1109/hri.2016.7451820

[B72] Olsen D. R. Wood S. B. (2004). “Fan-out: Measuring Human Control of Multiple Robots,” in Human Factors in Computing Systems (CHI). ACM, 231–238.

[B73] Peskoe-Yang L. (2019). Paris Firefighters Used This Remote-Controlled Robot to Extinguish the Notre Dame Blaze. IEEE Spectrum. April 22.

[B74] Pfeiffer C. Scaramuzza D. (2021). Human-piloted Drone Racing: Visual Processing and Control. IEEE Robot. Autom. Lett. 6 (2), 3467–3474. 10.1109/lra.2021.3064282

[B75] Quigley M. Goodrich M. A. Beard R. W. (2004). “Semi-autonomous Human-UAV Interfaces for Fixed-wing Mini-UAVs,” in 2004 IEEE/RSJ International Conference on Intelligent Robots and Systems (IROS) IEEE, 3, 2457–2462.

[B76] Rae I. Neustaedter C. (2017). “Robotic Telepresence at Scale,” in Conference on Human Factors in Computing Systems. ACM, 313–324. 10.1145/3025453.3025855

[B77] Rakita D. Mutlu B. Gleicher M. (2017). “A Motion Retargeting Method for Effective Mimicry-Based Teleoperation of Robot Arms,” in Proceedings of the 2017 ACM/IEEE International Conference on Human-Robot Interaction, Vienna, Austria, 6-9 March 2017, 361–370. 10.1145/2909824.3020254

[B78] Rakita D. Mutlu B. Gleicher M. (2018). “An Autonomous Dynamic Camera Method for Effective Remote Teleoperation. ACM/IEEE,” in Human-Robot Interaction, 325–333. 10.1145/3171221.3171279

[B79] Rakita D. Mutlu B. Gleicher M. Hiatt L. M. (2019b). Shared Control-Based Bimanual Robot Manipulation. Sci. Robot. 4, 30. 10.1126/scirobotics.aaw0955 33137728

[B80] Rakita D. Mutlu B. Gleicher M. (2019a). “Remote Telemanipulation with Adapting Viewpoints in Visually Complex Environments,” in Robotics: Science and Systems XV. Belfast, Ireland. 10.15607/rss.2019.xv.068

[B81] Rea D. J. Hanzaki M. R. Bruce N. Young J. E. (2017a). “Tortoise and the Hare Robot Slow and Steady Almost Wins the Race, but Finishes More Safely,” in Robot and Human Interactive Communication RO-MAN (IEEE), 1–6. 10.1109/roman.2017.8172418

[B82] Rea D. J. (2020). Now You’re Teleoperating with Power: Learning from Video Games to Improve Teleoperation Interfaces. Winnipeg, Canada: University of Manitoba.

[B83] Rea D. J. Seo S. H. Bruce N. Young J. E. (2017b). “Movers, Shakers, and Those Who Stand Still: Visual Attention-Grabbing Techniques in Robot Teleoperation,” in Proceedings of the 2017 ACM/IEEE International Conference on Human-Robot Interaction. ACM/IEEE, 398–407.

[B84] Rea D. J. Young J. E. (2019a). “Backseat Teleoperator : Affective Feedback with On-Screen Agents to Influence Teleoperation,” in Proceedings of the 2019 ACM/IEEE International Conference on Human-Robot Interaction, Daegu, Korea (South), 11-14 March 2019, 19–28. 10.1109/hri.2019.8673014

[B85] Rea D. J. Young J. E. (2018). “It’s All in Your Head: Using Priming to Shape an Operator’s Perceptions and Behavior during Teleoperation,” in Proceedings of the 2018 ACM/IEEE International Conference on Human-Robot Interaction, Chicago, IL, USA, 5-8 March 2018, 32–40.

[B86] Rea D. J. Young J. E. (2019b). “Methods and Effects of Priming a Teloperator’s Perception of Robot Capabilities,” in 2019 14th ACM/IEEE International Conference on Human-Robot Interaction (HRI), Daegu, Korea (South), 11-14 March 2019, 739–741.

[B87] Rea D. J. Seo S. H. Young J. E. (2020). Social Robotics for Nonsocial Teleoperation: Leveraging Social Techniques to Impact Teleoperator Performance and Experience. Curr. Robot Rep. 1 (4), 287–295. 10.1007/s43154-020-00020-7

[B88] Reddy S. Dragan A. D. Levine S. (2018). “Shared Autonomy via Deep Reinforcement Learning,” in Robotics: Science and Systems (RSS). New York, NY, United States. 10.15607/rss.2018.xiv.005

[B89] Reveleau A. Ferland F. Labbé M. Létourneau D. Michaud F. (2015). Visual Representation of Sound Sources and Interaction Forces in a Teleoperation Interface for a Mobile Robot. J. Human-Robot Interaction 4 (2), 1. 10.5898/jhri.4.2.reveleau

[B90] Richer J. Drury J. L. (2006). “A Video Game-Based Framework for Analyzing Human-Robot Interaction: Characterizing Interface Design in Real-Time Interactive Multimedia Applications,” in Human-Robot Interaction. ACM, 266–273.

[B91] Saakes D. Choudhary V. Sakamoto D. Inami M. Igarashi T. (2013). “A Teleoperating Interface for Ground Vehicles Using Autonomous Flying Cameras,” in International Conference on Artificial Reality and Telexistence (ICAT) Tokyo, Japan: IEEE, 13–19. 10.1109/icat.2013.6728900

[B92] Sakamoto D. Honda K. Inami M. Igarashi T. (2009). “Sketch and Run: a Stroke-Based Interface for home Robots,” in Conference on Human Factors in Computing Systems. ACM, 197–200.

[B93] Sarter N. B. Woods D. D. (1995). How in the World Did We Ever Get into that Mode? Mode Error and Awareness in Supervisory Control. Hum. Factors 37 (1), 5–19. 10.1518/001872095779049516

[B94] Schilling K. Roth H. Lieb R. (1997). “Teleoperations of Rovers. From Mars to Education,” in International Symposium on Industrial Electronics. IEEE, 257–262.

[B95] Seo S. H. Rea D. J. Wiebe J. Young J. E. (2017a). “Monocle: Interactive Detail-In-Context Using Two Pan-And-Tilt Cameras to Improve Teleoperation Effectiveness,” in 2017 26th IEEE International Symposium on Robot and Human Interactive Communication (RO-MAN), 962–967. 10.1109/roman.2017.8172419

[B96] Seo S. H. Young J. E. Irani P. (2020). “How Are Your Robot Friends Doing? A Design Exploration of Graphical Techniques Supporting Awareness of Robot Team Members in Teleoperation,” in International Journal of Social Robotics. Springer.

[B97] Seo S. H. Young J. E. Irani P. (2017b). “Where Are the Robots? In-Feed Embedded Techniques for Visualizing Robot Team Member Locations,” in Robot and Human Interactive Communication. IEEE, 522–527.

[B98] Settimi A. Pavan C. Varricchio V. Ferrati M. Mingo Hoffman E. Rocchi A. (2014). “A Modular Approach for Remote Operation of Humanoid Robots in Search and rescue Scenarios,” in Modelling and Simulation for Autonomous Systems (MESAS), 8906. Springer, 200–205. 10.1007/978-3-319-13823-7_18

[B99] Singh A. Seo S. H. Hashish Y. Nakane M. Young J. E. Bunt A. (2013). “An Interface for Remote Robotic Manipulator Control that Reduces Task Load and Fatigue,” in IEEE International Symposium on Robot and Human Interactive Communication (RO-MAN). IEEE, 738–743. 10.1109/roman.2013.6628401

[B100] Small N. Lee K. Mann G. (2018). An Assigned Responsibility System for Robotic Teleoperation Control. Int. J. Intell. Robot Appl. 2 (1), 81–97. 10.1007/s41315-018-0043-0 29577073 PMC5854743

[B101] Squire P. N. Parasuraman R. (2010). Effects of Automation and Task Load on Task Switching during Human Supervision of Multiple Semi-autonomous Robots in a Dynamic Environment. Ergonomics 53 (8), 951–961. 10.1080/00140139.2010.489969 20658389

[B102] Tadokoro S. (Editor) (2019). Disaster Robotics (Springer).

[B103] Steinfeld A. Fong T. Kaber D. Lewis M. Scholtz J. Schultz A. (2006). “Common Metrics for Human-Robot Interaction,” in Proceeding of the 1st ACM SIGCHI/SIGART Conference on Human-Robot Interaction - HRI ’06. ACM, 33. 10.1145/1121241.1121249

[B104] Suarez Fernandez R. A. Luis Sanchez-Lopez J. Sampedro C. Bavle H. Molina M. Campoy P. (2016). “Natural User Interfaces for Human-Drone Multi-Modal Interaction,” in 2016 International Conference on Unmanned Aircraft Systems (ICUAS). IEEE, 1013–1022. 10.1109/icuas.2016.7502665

[B105] Sugiura Y. Sakamoto D. Withana A. Inami M. Igarashi T. (2010). “Cooking with Robots,” in Proceedings of the 28th international conference on Human factors in computing systems - CHI ’10, Atlanta Georgia USA, April 10 - 15, 2010, 2427. 10.1145/1753326.1753693

[B106] Szafir D. Szafir D. A. (2021). “Connecting Human-Robot Interaction and Data Visualization,” in ACM/IEEE International Conference on Human-Robot Interaction. ACM, 281–292. 10.1145/3434073.3444683

[B107] Teng W. C. Kuo Y. C. Yanu Tara R. (2013). “A Teleoperation System Utilizing Saliency-Based Visual Attention,” in IEEE International Conference on Systems, Man, and Cybernetics. IEEE, 139–144. 10.1109/smc.2013.31

[B108] Tsui K. M. Adam Norton D. J. B. McCann E. Medvedev M. S. Yanco H. A. (2013). “Design and Development of Two Generations of Semi-autonomous Social Telepresence Robots,” in 2013 IEEE Conference on Technologies for Practical Robot Applications (TePRA). IEEE.

[B109] Tsui K. M. Dalphond J. M. Brooks D. J. Medvedev M. S. McCann E. Allspaw J. (2015). Accessible Human-Robot Interaction for Telepresence Robots: A Case Study. Paladyn, J. Behav. Robotics 6 (1), 1–29. 10.1515/pjbr-2015-0001

[B110] Turchetti G. Palla I. Pierotti F. Cuschieri A. (2012). Economic evaluation of da Vinci-assisted robotic surgery: A systematic review. Surg. Endosc. 26 (3), 598–606. 10.1007/s00464-011-1936-2 21993935

[B111] Valiton A. Li Z. (2020). “Perception-Action Coupling in Usage of Telepresence Cameras,” in Proceedings - IEEE International Conference on Robotics and Automation. IEEE, 3846–3852. 10.1109/icra40945.2020.9197578

[B112] Wainer J. Feil-Seifer D. J. Shell D. A. Matarić M. J. (2006). “The Role of Physical Embodiment in Human-Robot Interaction,” in Proceedings - IEEE International Workshop on Robot and Human Interactive Communication, 117–122. 10.1109/roman.2006.314404

[B113] Wang J. Lewis M. (2007). “Human Control for Cooperating Robot Teams,” in HRI 2007 - Proceedings of the 2007 ACM/IEEE Conference on Human-Robot Interaction - Robot as Team Member. ACM/IEEE, 9–16. 10.1145/1228716.1228719

[B114] Wong C. Y. Seet G. (2017). Workload, Awareness and Automation in Multiple-Robot Supervision. Int. J. Adv. Robotic Syst. 14 (3), 1–16. 10.1177/1729881417710463

[B115] Yanco H. A. Norton A. Ober W. Shane D. Skinner A. Vice J. (2015). Analysis of Human-Robot Interaction at the DARPA Robotics Challenge Trials. J. Field Robotics 32 (3), 420–444. 10.1002/rob.21568

[B116] Yanco H. A. Drury J. (2004b). Classifying Human-Robot Interaction: An Updated Taxonomy. IEEE Int. Conf. Syst. Man Cybernetics 3, 2841–2846. 10.1109/ICSMC.2004.1400763

[B117] Yanco H. A. Drury J. L. Scholtz J. (2004). Beyond Usability Evaluation : Analysis of Human-Robot Interaction at a Major Robotics Competition. Human Comput. Inter. ACM 19–1, 931414160. 10.1207/s15327051hci1901&2_6

[B118] Yanco H. A. Drury J. (2004a). ““Where Am I?” Acquiring Situation Awareness Using a Remote Robot Platform,” in IEEE Systems, Man and Cybernetics. IEEE, 2835–2840.

[B119] Yang J. Kamezaki M. Sato R. Iwata H. Sugano S. (2015). “Inducement of Visual Attention Using Augmented Reality for Multi-Display Systems in Advanced Tele-Operation,” in 2015 IEEE/RSJ International Conference on Intelligent Robots and Systems (IROS), Hamburg, Germany, 28 Sept.-2 Oct. 2015, 5364–5369.

[B120] Young J. E. Kamiyama Y. Reichenbach J. Igarashi T. Sharlin E. (2011). How to Walk a Robot: A Dog-Leash Human-Robot Interface RO-MAN. IEEE, 376–382.

[B121] Young S. N. Peschel J. M. (2020). Review of Human-Machine Interfaces for Small Unmanned Systems with Robotic Manipulators. IEEE Trans. Human-mach. Syst. 50 (2), 131–143. 10.1109/thms.2020.2969380

